# Geographic and socioeconomic factors associated with leprosy treatment default: An analysis from the 100 Million Brazilian Cohort

**DOI:** 10.1371/journal.pntd.0007714

**Published:** 2019-09-06

**Authors:** Kaio Vinicius Freitas de Andrade, Joilda Silva Nery, Julia Moreira Pescarini, Anna Ramond, Carlos Antônio de Souza Teles Santos, Maria Yury Ichihara, Maria Lucia Fernandes Penna, Elizabeth B. Brickley, Laura C. Rodrigues, Liam Smeeth, Mauricio L. Barreto, Susan Martins Pereira, Gerson Oliveira Penna

**Affiliations:** 1 Centre for Data and Knowledge Integration for Health (CIDACS), Oswaldo Cruz Foundation (FIOCRUZ), Salvador, Brazil; 2 Institute of Collective Health (ISC), Federal University of Bahia (UFBA), Salvador, Brazil; 3 Department of Health, State University of Feira de Santana (UEFS), Feira de Santana, Brazil; 4 Department of Infectious Disease Epidemiology, London School of Hygiene & Tropical Medicine, London, United Kingdom; 5 Department of Epidemiology and Biostatistics, Federal University Fluminense, Rio de Janeiro, Brazil; 6 Department of Non-communicable Disease Epidemiology, London School of Hygiene & Tropical Medicine, London, United Kingdom; 7 Tropical Medicine Centre, University of Brasília, Fiocruz School of Government, Fiocruz Brasília, Brazil; Hospital Infantil de Mexico Federico Gomez, UNITED STATES

## Abstract

**Background:**

Although leprosy is largely curable with multidrug therapy, incomplete treatment limits therapeutic effectiveness and is an important obstacle to disease control. To inform efforts to improve treatment completion rates, we aimed to identify the geographic and socioeconomic factors associated with leprosy treatment default in Brazil.

**Methodology/Principal findings:**

Using individual participant data collected in the Brazilian national registries for social programs and notifiable diseases and linked as part of the 100 Million Brazilian Cohort, we evaluated the odds of treatment default among 20,063 leprosy cases diagnosed and followed up between 2007 and 2014. We investigated geographic and socioeconomic risk factors using a multivariate hierarchical analysis and carried out additional stratified analyses by leprosy subtype and geographic region. Over the duration of follow-up, 1,011 (5.0%) leprosy cases were observed to default from treatment. Treatment default was markedly increased among leprosy cases residing in the North (OR = 1.57; 95%CI 1.25–1.97) and Northeast (OR = 1.44; 95%CI 1.17–1.78) regions of Brazil. The odds of default were also higher among cases with black ethnicity (OR = 1.29; 95%CI 1.01–1.69), no income (OR = 1.41; 95%CI 1.07–1.86), familial income ≤ 0.25 times Brazilian minimum wage (OR = 1.42; 95%CI 1.13–1.77), informal home lighting/no electricity supply (OR = 1.53; 95%CI 1.28–1.82), and household density of > 1 individual per room (OR = 1.35; 95%CI 1.10–1.66).

**Conclusions:**

The findings of the study indicate that the frequency of leprosy treatment default varies regionally in Brazil and provide new evidence that adverse socioeconomic conditions may represent important barriers to leprosy treatment completion. These findings suggest that interventions to address socioeconomic deprivation, along with continued efforts to improve access to care, have the potential to improve leprosy treatment outcomes and disease control.

## Author summary

While the leprosy new case detection has been decreasing worldwide since the introduction of multidrug therapy (MDT) in the 1980s, treatment default remains an important risk factor for leprosy-associated disability and an obstacle to disease control and elimination. Treatment default occurs when an individual with leprosy does not take the prescribed number of doses required for treatment with MDT. We hypothesized that the frequency of defaulting may be influenced by geographic factors, especially as related to access to care, and socioeconomic factors, such as income, education, and household living conditions. To test this hypothesis, we investigated geographic and socioeconomic factors associated with leprosy treatment default among 20,063 new leprosy cases followed as part of the 100 Million Brazilian Cohort between 2007 and 2014. In total, 5.0% of the leprosy patients defaulted from MDT. Among the associated factors, we found that having residency in the North and Northeast of Brazil, black ethnicity, low familial income, lack of formal electricity, and a high household density were associated with higher odds of leprosy treatment default. Overall, these findings highlight the need for tailoring MDT strategies for vulnerable populations in high-burden communities and suggest that social policies aiming to alleviate poverty should be investigated as potential tools for improving leprosy treatment completion.

## Introduction

Leprosy, also known as Hansen’s disease, is a chronic and potentially disabling infectious disease caused by *Mycobacterium leprae* that primarily affects peripheral nerves and skin [1, 2]. Since the introduction of multidrug therapy (MDT) in 1982, the global burden of leprosy has been significantly decreasing [3, 4, 5]. In 2017, the World Health Organization (WHO) reported 210,671 new cases of leprosy, including 26,875 from Brazil [3].

In endemic countries, treatment defaulting is still an important obstacle to effective leprosy control and elimination [6, 7]. Specifically, interruptions and defaults from treatment may result in incomplete cures and persisting sources of infection in affected communities. Concerns have also been raised that patient non-adherence to MDT has the potential to contribute to drug resistance [8]. Further, delays in leprosy diagnosis and inadequate treatment may lead to irreversible physical disabilities that can cause stigma and social disadvantages in affected people [4].

Leprosy patients are grouped for treatment purposes according to their number of skin lesions: cases are classified as paucibacillary (PB) if they have up to five skin lesions and multibacillary (MB) in the presence of more than five skin lesions [1, 2]. The classification of PB versus MB defines the nature and duration of the treatment regimen. Broadly, the term defaulting from treatment describes when an individual with leprosy does not complete the full MDT treatment despite repeated efforts from health services to ensure treatment completion [2].

As recently systematically reviewed by Girão and colleagues (2013), there exists a limited evidence base regarding the determinants of leprosy treatment default [9]. Current evidence suggests leprosy treatment default may be influenced by both personal characteristics (e.g., quality of life, socioeconomic position) and medical factors (e.g., treatment regimen and guidance, clinic distance, drug shortages) [9]. Further, some poverty-related variables, including a low number of rooms per household and low familial income, have also been associated with leprosy treatment default in one population-based study in central Brazil [6].

Utilizing individual participant data from more than 20,000 leprosy cases followed up between 2007–2014 as part of the 100 Million Brazilian Cohort, this study used a hierarchical approach to investigate the association of geographic and socioeconomic factors with (i) overall leprosy treatment default, (ii) leprosy treatment default in PB and MB subtypes, and (iii) leprosy treatment default within Brazilian geographic regions.

## Methods

### Study design

The cohort used in this study was derived from the 100 Million Brazilian Cohort created by the Centre for Data and Knowledge Integration for Health at Oswaldo Cruz Foundation (CIDACS/FIOCRUZ, Salvador, Bahia, Brazil). The aim of the 100 Million Brazilian Cohort is to investigate the role of social determinants and the effects of social policies and programs on health, through the linkage of data from social programs with databases of health information systems [10].

The 100 Million Brazilian Cohort was built using the baseline information of the national registry for social programs, *Cadastro Único* (CadÚnico), from 2001 to 2015. CadÚnico contains administrative records of all families applying for social programs in Brazil. To date, the 100 Million Brazilian Cohort includes socioeconomic data on over 114 million individuals. The individual records were linked with nationwide health datasets, including the 2007–2014 leprosy registries from the ‘*Sistema de Informação de Agravos de Notificação*’ (SINAN-leprosy), through a deterministic algorithm, using the CIDACS-RL tool (https://gitHub.com/gcgbarbosa/cidacs-rl). The specific variables used to match both datasets were patients’ name, date of birth, sex, mother’s name and municipality of residence. To assess the accuracy of data linkage, we carried out a manual analysis with a random sample of 10,000 pairs. For a cutoff of 0.93, sensitivity was 0.91 (95% CI 0.90–0.92) and specificity was 0.89 (0.88–0.90). The full linked dataset was de-identified to ensure anonymity/confidentiality of personal information and was made available for research from January 2018 (https://hdl.handle.net/20.500.12196/FK2/FNMRCA). CIDACS implemented strong data security rules to control access, use, and data privacy and integrity.

### Study population

**Fig 1:**
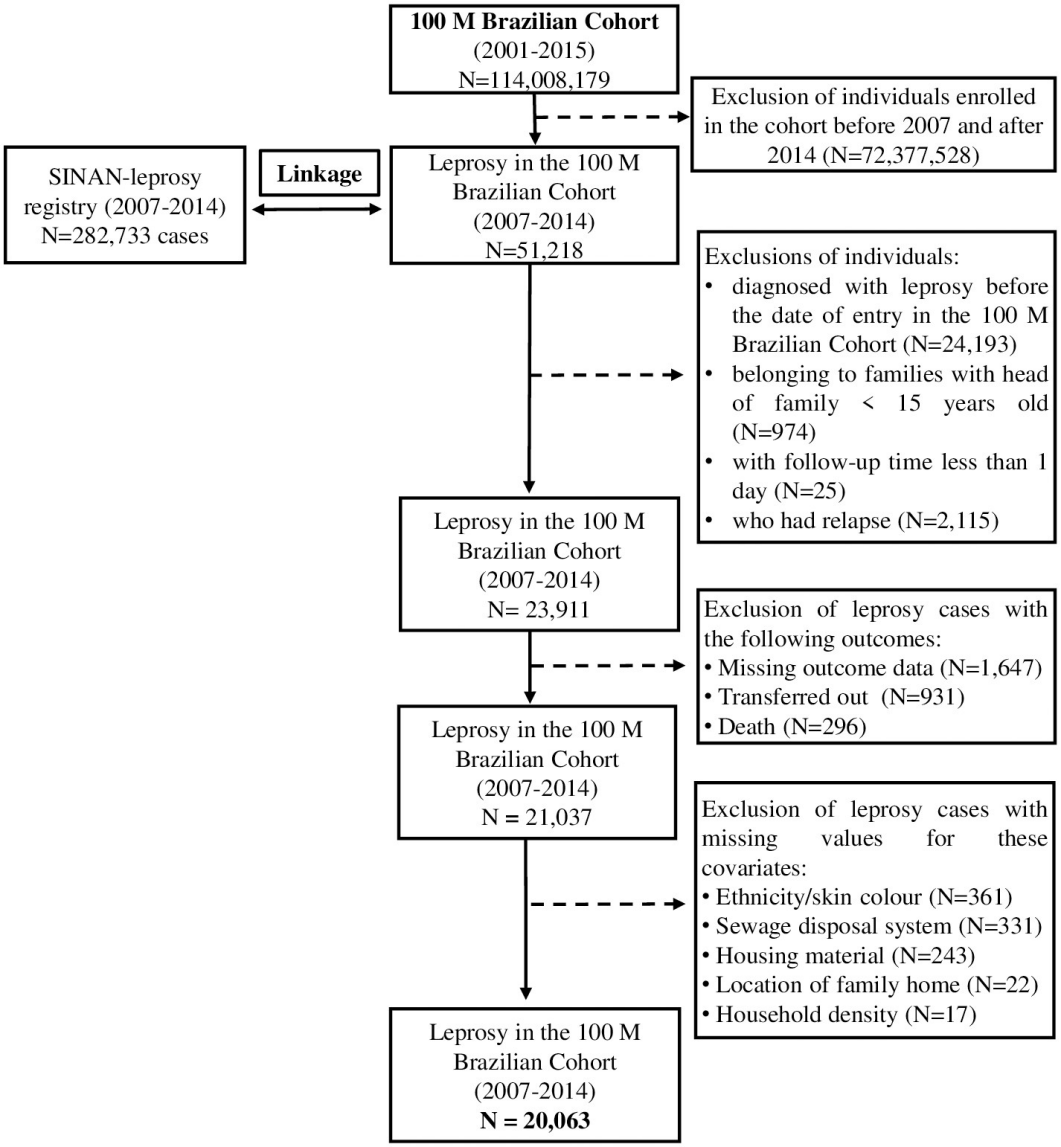
Study population selection flowchart from the 100 Million Brazilian Cohort.

The final subset of the 100 Million Brazilian Cohort used in this study was restricted to individuals who were diagnosed with leprosy after enrolment in the cohort between 1 January 2007 and 31 December 2014. Family units within the dataset included at least one member aged over 15 years old, with the oldest member of each family designated as the ‘head of the family.’ Individuals were excluded if they: (i) were diagnosed with leprosy prior enrollment in the cohort, (ii) belonged to family units without one member aged over 15 (i.e., children who were registered separately from their original families were excluded from the study), (iii) had less than 1 day of follow-up on SINAN-leprosy, and (iv) were relapsed cases. Records with missing data on the study outcome and/or covariates were also excluded. Only for the covariates of schooling and employment (with missing values ≥10%), missing information were considered as an additional category (Fig 1).

### Conceptual model

**Fig 2:**
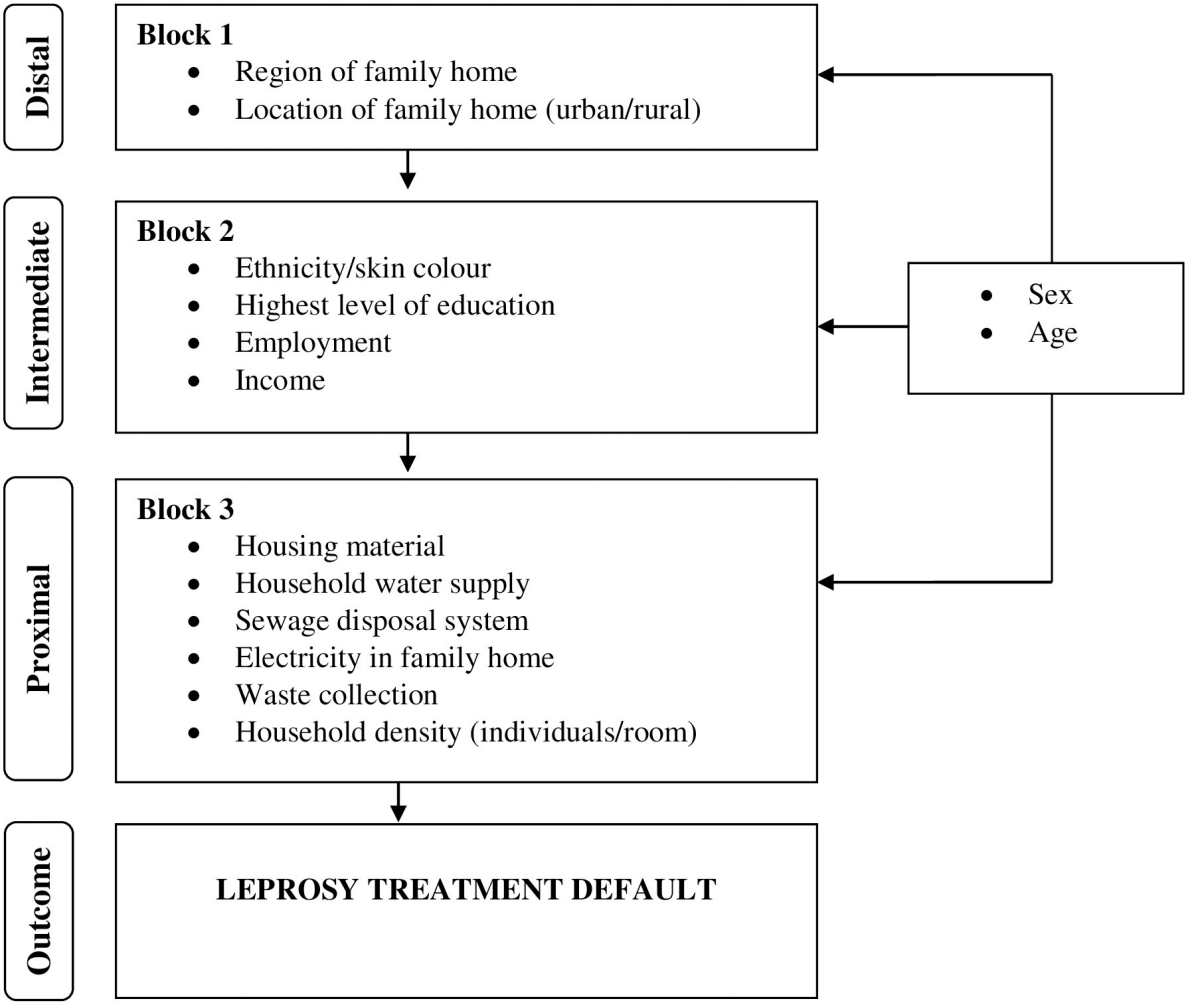
Hierarchical model for assessing geographic and socioeconomic factors associated with leprosy treatment default in Brazil.

We constructed a theoretical framework in which variables were grouped in three levels and blocks according to a predefined hierarchy represented by the conceptual framework shown in Fig 2 [11]. The distal level included geographic variables: region of residence in the country and location of family home (i.e., urban versus rural). The intermediate level was related to the socioeconomic position in the community and included: ethnicity/skin colour (according to the self-identified classification used in the Brazilian census) [12], the highest level of education, employment and per capita family income (i.e., presented relative to Brazilian minimum wage).

For individuals aged less than 18 years, schooling and occupation of the ‘head of the family’ were used as a proxy indicator. The proximal level comprised a set of variables related to household conditions experienced at the family level and included: housing material, household water supply, sewage disposal system, the source of home lighting, waste collection and household density (i.e., individuals per room). Because sex and age were considered as confounders *a priori*, they were included in all analyses.

The study outcome was leprosy treatment default defined as a binary variable (i.e., default versus cure) among newly detected leprosy cases [2]. For PB cases, treatment completion comprises 6 monthly doses of MDT until 9 months. For MB cases, treatment completion comprises 12 monthly doses of MDT until 18 months. The term ‘defaulter’ refers to leprosy patients who does not complete these full MDT treatment regimens (PB patients who does not attend treatment for more than 3 months and MB patients for more than 6 months), even after repeated efforts of health professionals to tracking patients for treatment completion [2].

### Statistical analysis

We conducted a descriptive analysis assessing the role of each geographic and socioeconomic variable on the study outcome in bivariate analyses. Then, in a multivariate analysis, blocks of variables from distal to proximal levels were added in a sequence following a hierarchical approach [11] as shown in the conceptual framework. The study outcome was analysed using logistic regression with cluster-robust standard errors to account for familial clustering of covariates. Because of the low prevalence of this study outcome (i.e., in less than 10%), the odds ratio (OR) estimates and their 95% confidence intervals (CI) provided a close approximation of the risk ratios [13].

An effect-decomposition strategy was applied to fit three logistic regression models (A, B, and C) by including step-by-step blocks of variables [11]. Variables in each block that were associated with leprosy treatment default at a significance threshold of *P*<0.10 were included in the next level model, with all models adjusting for sex and age.

As a secondary analysis, we investigated the associations by leprosy subtype and across geographic regions. Because MB leprosy cases have been reported to have higher rates of treatment default and onward transmission than PB cases [1, 7, 14–17], we compared the associations by leprosy subtype (i.e., PB versus MB). In addition, reflecting the important regional differences in social inequalities in Brazil, we performed analyses stratified by region (North, Northeast, Midwest and South/Southeast) [12].

All *P*-values were calculated for 2-sided statistical tests, and all analyses were performed using Stata, version 15.0 (StataCorp LLC, College Station, Texas, USA).

### Ethics considerations

No personally identifiable information was included in the datasets used for analysis. Further, all data included in this study were stored on secured servers within CIDACS with strict access restrictions.

This study was performed under the international (Helsinki), Brazilian and United Kingdom research regulations and was approved by three ethics committee of research: (i) University of Brasília (UnB) (protocol n° 1.822.125), (ii) *Instituto Gonçalo Moniz*/FIOCRUZ (protocol n° 1.612.302) and (iii) London School of Hygiene and Tropical Medicine’s Research Committee (protocol n° 10580–1).

## Results

Among 20,063 new cases of leprosy, 1,011 (5.0%) defaulted from treatment. The percentage of default varied from 6.4% in 2007 to 5.4% in 2014. Approximately half of the leprosy cases (N = 10,101, 50.4%) were female. The median age was 34.9y (IQ 24.6–52.5y), and 17,179 (85.6%) were aged 15y or more. The proportion of children less than 15 years (14.6%) was nearly 2-fold the average of new child cases in Brazil (7.3% of all new leprosy cases during 2007–2014) [18]. The median per capita income in US dollar (USD) was 34.0 (IQ 16.6–89.3). 13,063 (65.1%) were residents in the Northeast and North regions, 16,050 (80.0%) lived in an urban setting and 14,511 (72.3%) self-identified as having a ‘pardo’ (mixed) ethnicity. 10,858 individuals (54.1%) had up to 5 years of schooling, 11,080 (55.2%) had a per capita familial income up to a quarter of the Brazilian minimum wage, and 9,030 (45.0%) were unemployed or students. The majority of the leprosy cases lived in generally favourable household settings, with 13,956 (69.6%) residing in houses made of brick or cement, 13,797 (68.8%) accessing water supply networks, 16,166 (80.6%) accessing electricity through a home meter, 15,271 (76.1%) having public waste collection, and 15,267 (76.1%) residing in households with up to 1 individual per room. Nevertheless, 13,480 (67.2%) of the leprosy cases did not report access to improved sanitation (Table 1).

**Table 1:** Proportion of new leprosy cases (N = 20,063), proportion of defaulters in each subgroup and bivariate associations of geographic and socioeconomic factors with leprosy treatment default, Brazil, 2007–2014.

Variables	Total cases (N = 20,063)		Defaulters (N = 1,011)		Crude OR (95% CI)
	n	%*	n	%**	
**Sex**					
Female	10,101	50.4	545	5.4	1
Male	9,962	49.6	466	4.7	0.86 (0.76–1.00)
**Age**					
<15 years	2,884	14.4	110	3.8	1
≥15 years	17,179	85.6	901	5.2	1.40 (1.13–1.72)
**Distal variables**					
**Geographic region of family home**					
Northeast	8,428	42.0	447	5.3	1.48 (1.20–1.82)
North	4,635	23.1	266	5.7	1.61 (1.29–2.01)
Midwest	3,568	17.8	173	4.8	1.35 (1.06–1.71)
Southeast/South	3,432	17.1	125	3.6	1
**Location of family home**					
Urban	16,050	80.0	806	5.0	1
Rural	4,013	20.0	205	5.1	1.02 (0.87–1.19)
**Intermediate variables**					
**Ethnicity/skin colour**					
‘Pardo’ (Mixed/Brown)	14,511	72.3	733	5.0	1.12 (0.95–1.33)
Black	1,692	8.0	99	6.2	1.39 (1.07–1.79)
Other (White, Asian, Indigenous)	3,950	19.7	179	4.5	1
**Highest level of education**^†^					
No data (missing)	2,208	11.0	103	4.7	0.94 (0.71–1.24)
Pre-school/no education/illiterate	3,388	16.9	164	4.8	0.97 (0.75–1.26)
1–5 years	7,470	37.2	354	4.7	0.95 (0.76–1.20)
6–9 years	5,000	24.9	291	5.8	1.18 (0.94–1.50)
> 9 years	1,997	10.0	99	5.0	1
**Familial per capita income**^††^					
No income	2,164	10.8	117	5.4	1.52 (1.17–1.97)
0.1–0.25	11,080	55.2	619	5.6	1.57 (1.29–1.91)
0.26–0.5	3,294	16.4	147	4.5	1.24 (0.97–1.58)
> 0.5	3,525	17.6	128	3.6	1
Employment*					
Employed	8,950	44.6	466	5.2	1
Unemployed (not student)	4,999	24.9	236	4.7	0.90 (0.77–1.06)
Student	4,031	20.1	194	4.8	0.92 (0.77–1.09)
No data	2,083	10.4	115	5.5	1.06 (0.86–1.31)
**Proximal variables**					
**Housing material**					
Brick or cement	13,956	69.6	672	4.8	1
Wood, mud or similar	6,107	30.4	339	5.5	1.16 (1.01–1.33)
**Household water supply**					
Public network	13,797	68.8	673	4.9	1
Non-public network supply	6,266	31.2	338	5.4	1.11 (0.97–1.27)
**Source of home lighting**					
Home meter	16,166	80.6	746	4.6	1
Informal home lighting or no electricity	3,199	15.9	231	7.2	1.61 (1.38–1.88)
Community meter	698	3.5	34	4.9	1.06 (0.74–1.52)
**Waste collection**					
Public collection system	15,271	76.1	740	4.8	1
Informal waste collection	4,792	23.9	271	5.7	1.18 (1.02–1.36)
**Sewage disposal**					
Septic tank or open sewage	13,480	67.2	704	5.2	1.13 (0.98–1.29)
Public network	6,583	32.8	307	4.7	1
**Household density (individuals per room)**					
Up to 0.50	7,237	36.1	298	4.1	1
0.51–0.75	3,699	18.4	179	4.8	1.18 (0.98–1.43)
0.76–1.00	4,331	21.6	228	5.3	1.29 (1.08–1.54)
> 1.00	4,796	23.9	306	6.4	1.59 (1.34–1.87)

*Refers to the % of cases in each category of study variables among the total cases

**Refers to the % of defaulters in each category of study variables.

^†^Information on education and employment are reported at the individual level for adult participants (>18y) and for the oldest member of the family for participants aged under 18y

^††^in minimum wages in the Brazilian currency

In bivariate analyses, individuals from the North region were the most likely to default from leprosy treatment (OR = 1.61; 95%CI 1.29–2.01) as compared to South and Southeast residents (Table 1). Intermediate factors associated with defaulting were black ethnicity (OR = 1.39; 95%CI 1.07–1.79), no income (OR = 1.52; 95%CI 1.17–1.97) and per capita familial income up to a quarter of the minimum wage (OR = 1.57; 95%CI 1.29–1.91). Proximal factors associated with defaulting were: residency in accommodations constructed of wood and mud (OR = 1.16; 95%CI 1.01–1.33), informal home lighting or no electricity (OR = 1.61; 95%CI 1.38–1.88), no public waste collection (OR = 1.18; 95%CI 1.02–1.36), and household density between 0.75–1 (OR = 1.29; 95%CI 1.08–1.54) and > 1 individual per room (OR = 1.59; 95%CI 1.34–1.87) (Table 1).

In multivariate analysis, region of residence was also associated with treatment default in the distal model. Relative to the South/Southeast regions, the North, Northeast, and Midwest regions had increased odds of treatment default. Similar to the bivariate analyses, participants from the North region had the highest odds of defaulting from leprosy treatment in the full cohort (OR = 1.57; 95%CI 1.25–1.97) (Table 2).

**Table 2:** Results from multivariate hierarchical analysis of the association of geographic and socioeconomic factors with leprosy treatment default (N = 20,063), Brazil, 2007–2014.

Variable	MODEL A (Block 1)*		MODEL B (Blocks 1 and 2)**		MODEL C (Blocks 2 and 3)***	
	OR (95% CI)	P-value	OR (95% CI)	P-value	OR (95% CI)	P-value
**Sex**						
Male	0.87 (0.77–0.99)	0.035	0.88 (0.77–1.00)	0.055	0.88 (0.77–1.00)	0.047
Female	1		1		1	
**Age (per year)**	0.99 (0.99–1.00)	0.002	1.00 (0.99–1.00)	0.464	1.00 (0.99–1.00)	0.909
**Distal variables**						
**Region of family home**						
North	**1.57 (1.25–1.97)**	**<10**^**−3**^				
Northeast	**1.44 (1.17–1.78)**	**0.001**				
Midwest	**1.35 (1.06–1.72)**	**0.014**				
South/Southeast	1					
**Location of family home**						
Rural	0.97 (0.82–1.14)	0.691				
Urban	1					
**Intermediate variables**						
**Ethnicity/skin colour**						
Black			**1.29 (1.01–1.69)**	**0.045**		
‘Pardo’ (mixed/brown)			0.98 (0.82–1.16)	0.800		
Other (White, Asian, Indigenous)			1			
**Highest level of education**						
Pre-school/illiterate			0.99 (0.75–1.29)	0.926		
1–5 years			0.99 (0.78–1.25)	0.909		
6–9 years			1.17 (0.92–1.48)	0.188		
No data			0.89 (0.67–1.18)	0.421		
> 9 years			1			
**Familial per capita income**						
No income			**1.41 (1.07–1.86)**	**0.016**		
0.1–0.25			**1.42 (1.13–1.77)**	**0.002**		
0.26–0.5			1.18 (0.92–1.53)	0.189		
> 0.5			1			
**Employment**						
Unemployed (not student)			0.98 (0.82–1.18)	0.860		
Student			0.90 (0.75–1.09)	0.305		
No data			1.17 (0.94–1.46)	0.169		
Employed			1			
**Proximal variables**						
**Housing material**						
Wood, mud or others					0.97 (0.82–1.14)	0.679
Brick or cement					1	
**Household water supply**						
Non-public network supply					0.90 (0.76–1.07)	0.248
Public network					1	
**Sewage disposal**						
Septic tank or open sewage					0.96 (0.82–1.13)	0.661
Public network					1	
**Source of home lighting**						
Community meter					1.11 (0.77–1.61)	0.583
Informal home lighting or no electricity					**1.53 (1.28–1.82)**	**<10**^**−3**^
Home meter					1	
**Waste collection**						
Informal waste collection					1.00 (0.83–1.21)	0.993
Public collection system					1	
**Household density (individuals/room)**						
Up to 0.50					1	
0.51–0.75					1.10 (0.89–1.35)	0.371
0.76–1.00					1.18 (0.97–1.44)	0.100
> 1.00					**1.35 (1.10–1.66)**	**0.003**

* Covariates in model A were adjusted for sex and age;

** Covariates in model B were adjusted only for covariates from model A with p-value < 0.1, sex and age;

*** Covariates in model C were adjusted for covariates from model A and B with p-value < 0.1, sex and age.

Intermediate factors associated with treatment default in the full cohort included ethnicity and income. Participants who self-identified as black (OR = 1.29; 95%CI 1.01–1.69) and those with with 'no income'(OR = 1.41; 95%CI 1.07–1.86) and a per capita income up to 0.25 minimum wage (OR = 1.42; 95%CI 1.13–1.77) also had an increased probability of default from treatment. Of note, educational attainment and unemployment status were not associated with the odds of default (Table 2).

Among the proximal factors, no conventional home lighting or no electricity (OR = 1.53; 95%CI 1.28–1.82) and a household density greater than one person per room (OR = 1.35; 95%CI 1.10–1.66) were associated with increased probability of treatment default (Table 2). Housing material, water supply, sewage disposal, and waste collection were not associated with leprosy treatment default in the multivariate model.

**Fig 3:**
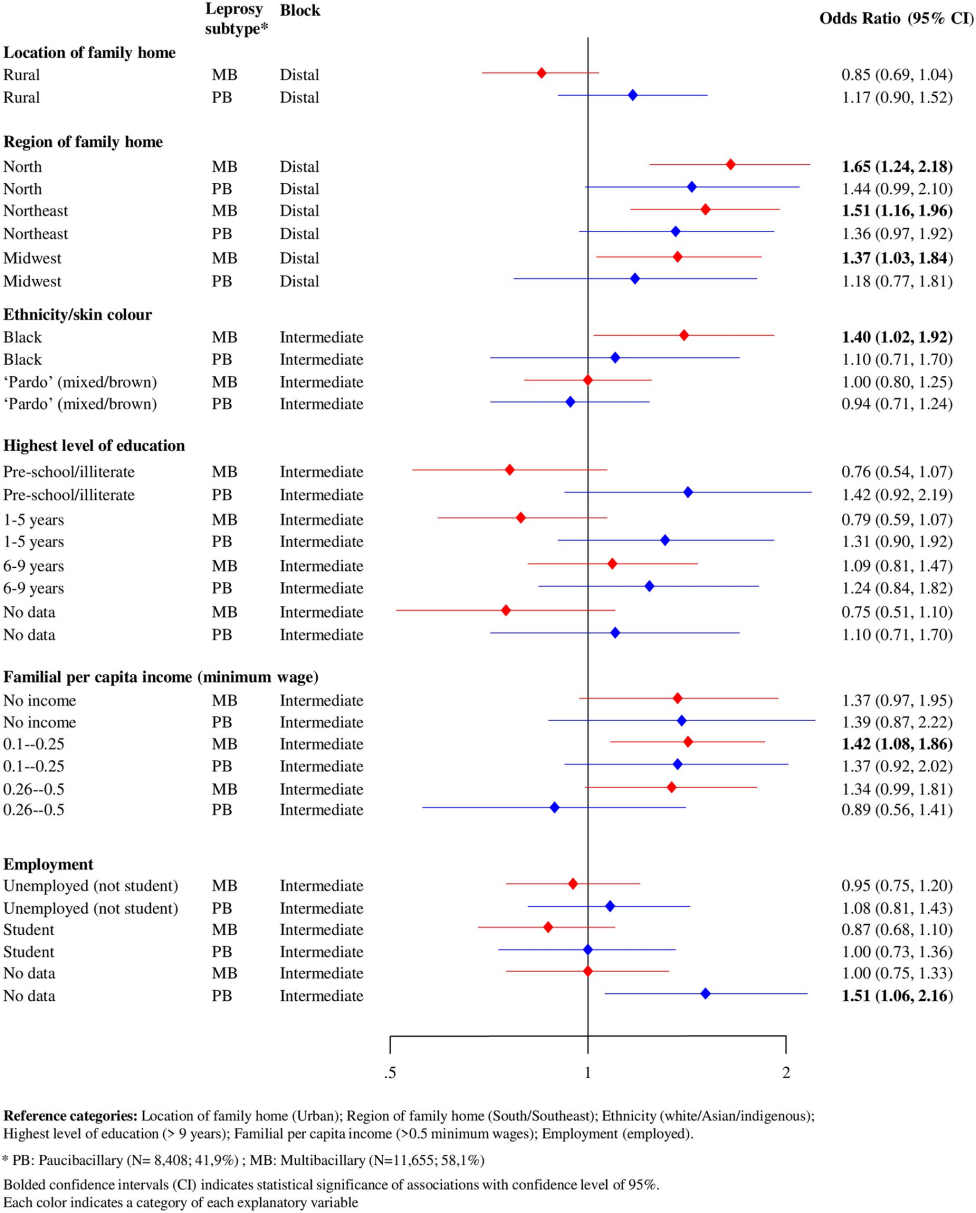
Forest plot of hierarchical association of distal and intermediate factors with leprosy treatment default (N = 20,063), stratified by leprosy subtype, Brazil, 2007–2014.

In the subgroup analyses of leprosy subtype, the directions of effect were broadly consistent across the PB and MB cases. The higher odds of treatment default among individuals from the North of Brazil remained consistent in this subgroup analyses, as residence in this region was most strongly associated with treatment default of MB leprosy cases (OR = 1.65 95%CI; 1.24–2.18). Regarding the intermediate factors, black ethnicity and income level up to 0.25 minimum wage were associated with treatment default only in MB patients (Fig 3).

**Fig 4:**
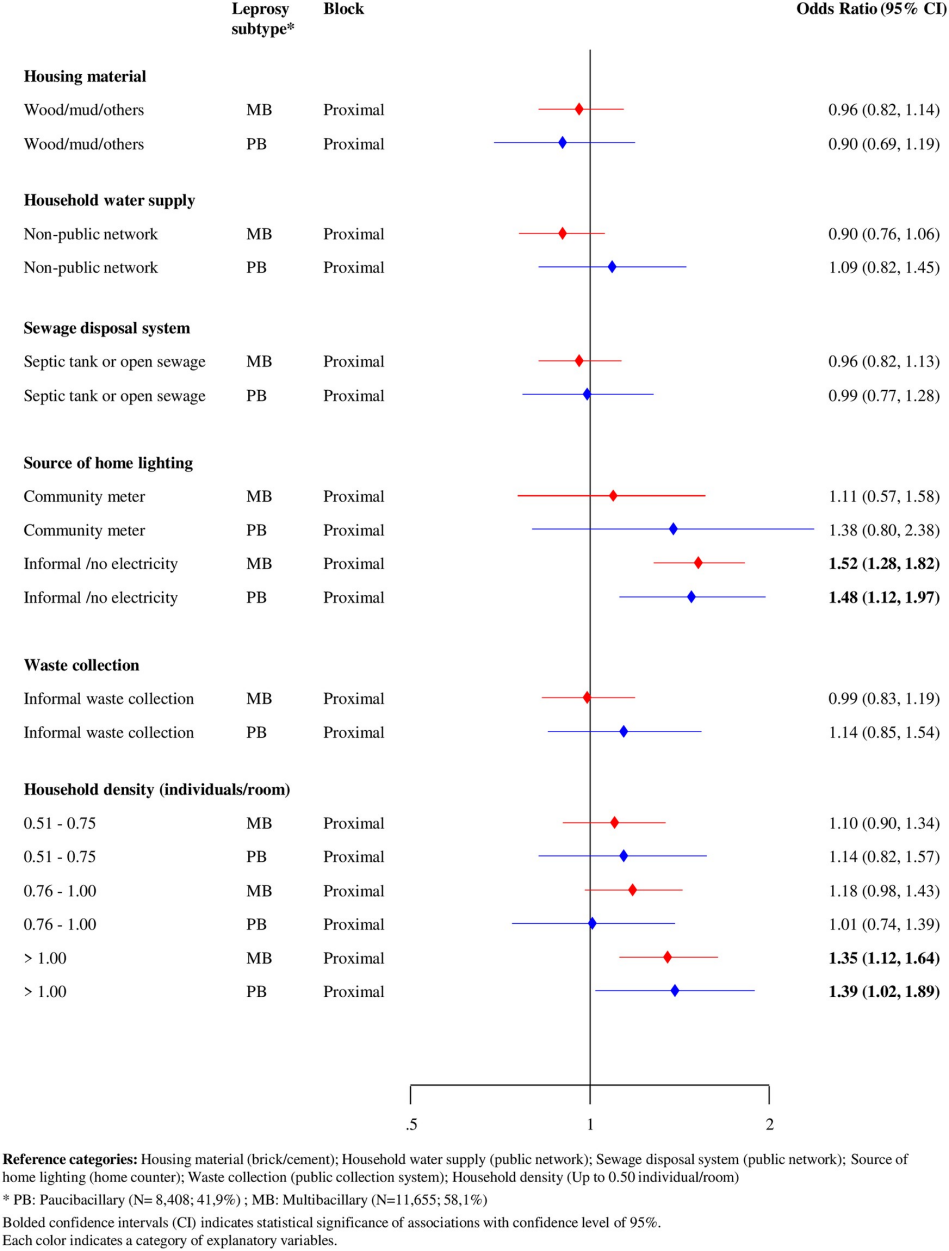
Forest plot of hierarchical association of proximal factors with leprosy treatment default (N = 20,063), stratified by leprosy subtype, Brazil, 2007–2014.

In relation to proximal factors, subgroup analyses of leprosy subtype showed that the use of informal home lighting or lack of electricity and a high household density (>1 individual peer room) remained associated with treatment default across both leprosy subtypes (PB and MB) (Fig 4).

**Fig 5:**
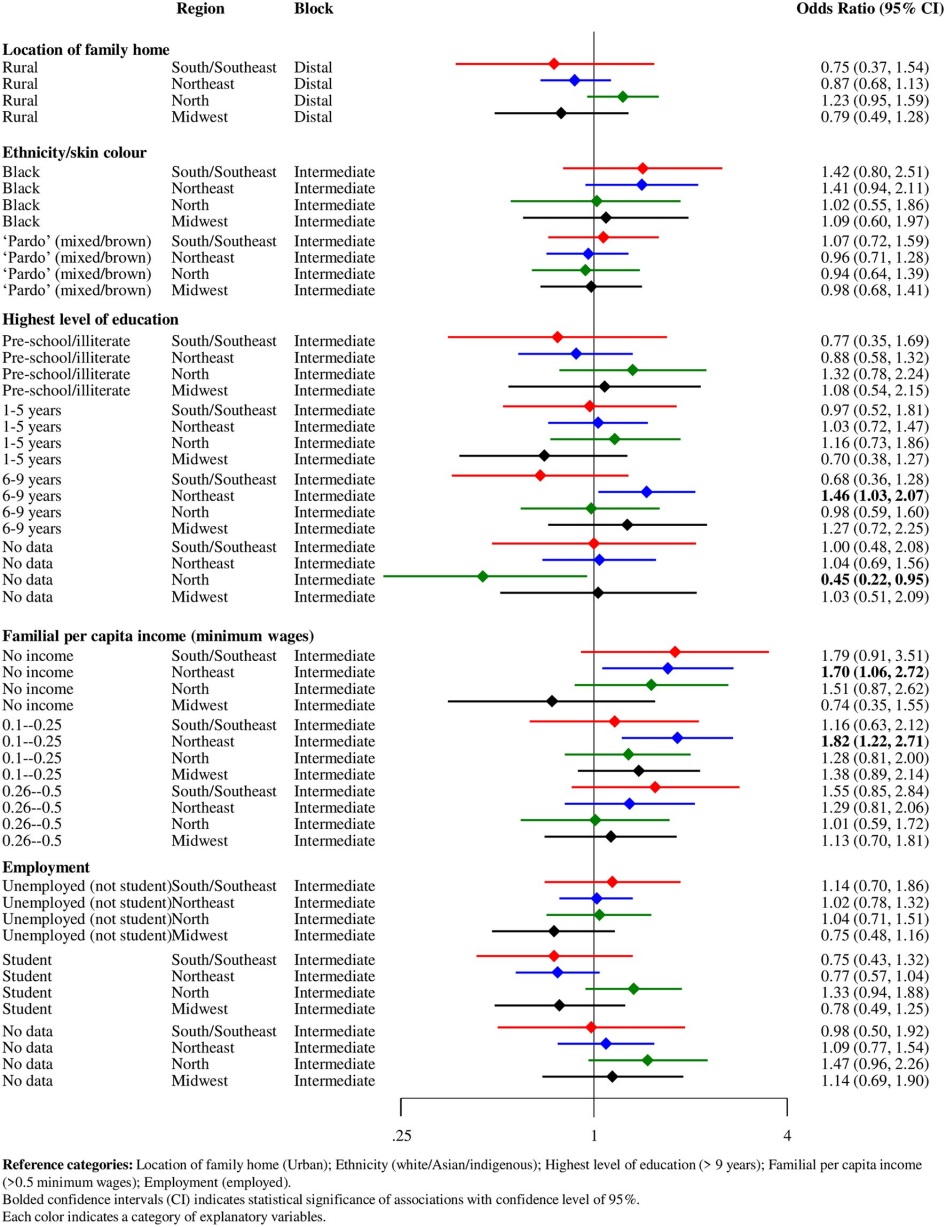
Forest plot of hierarchical association of distal and intermediate factors with leprosy treatment default (N = 20,063), stratified by Brazilian regions, Brazil, 2007–2014.

In the subgroup analyses by Brazilian regions, lowest income level (i.e., no income or income up to 0.25 minimum wage) was associated with odds of defaulting among residents in the Northeast region. An association between moderate educational attainment (i.e., 6–9 years) and treatment default was only found in the Northeast inhabitants (Fig 5).

**Fig 6:**
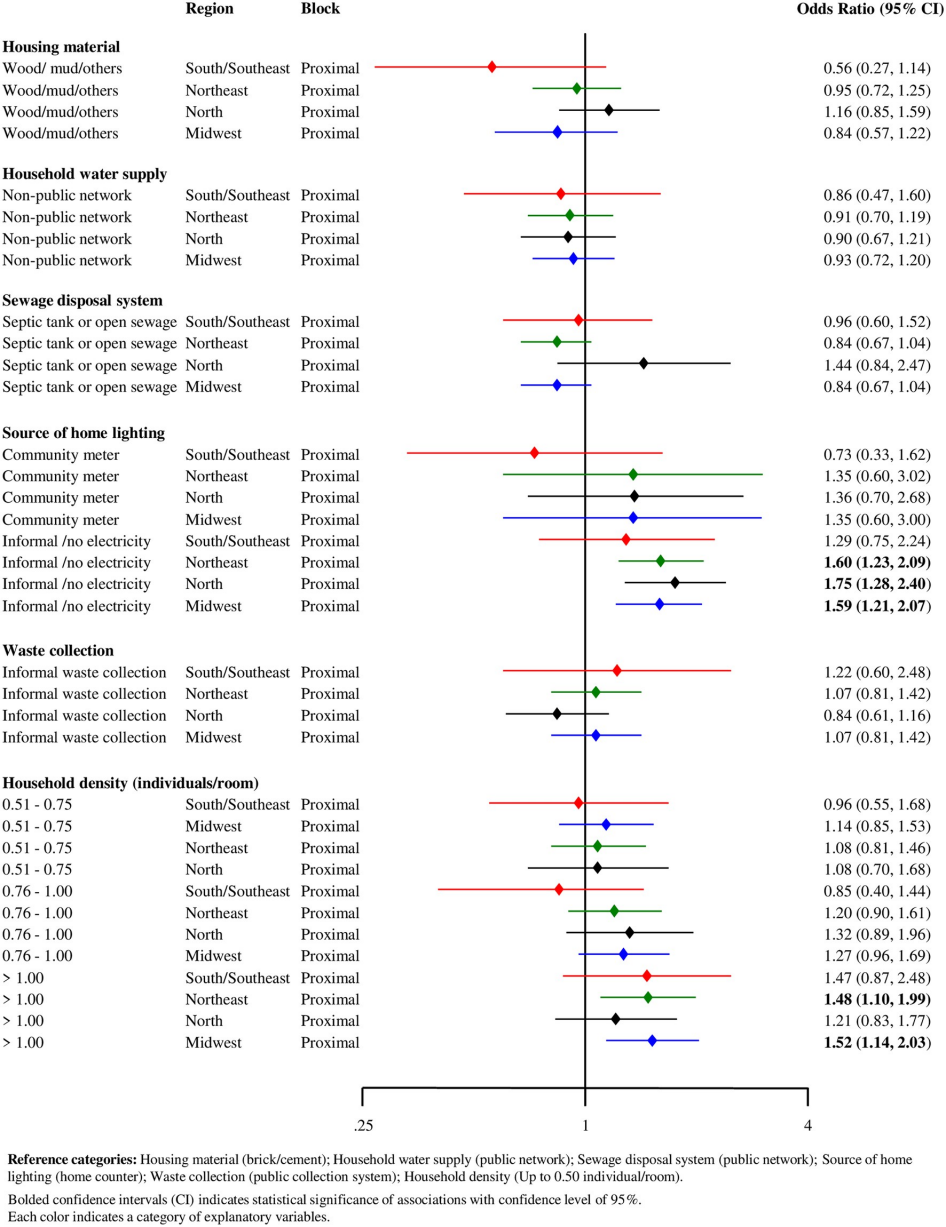
Forest plot of hierarchical association of proximal factors with leprosy treatment default (N = 20,063), stratified by Brazilian regions, Brazil, 2007–2014.

Subgroup analyses by region also revealed higher odds of treatment default associated with use of informal electricity or lack of electricity supply among residents in the North (OR = 1.75; 95%CI 1.28–2.40). Finally, a high household density (>1 individual per room) was associated with higher odds of treatment default of individuals living in the Midwest of Brazil (OR = 1.52; 95%CI 1.14–2.03) (Fig 6).

## Discussion

Using data from over 20,000 participants followed for up to 8 years, this cohort study is the largest to date investigating risk factors for leprosy treatment default (corresponding to 57.1% of the average of 35,130 new leprosy cases registered in all country during the same period) [18]. Our results revealed that individuals living in Brazilian regions carrying the highest leprosy burdens (i.e., North, Northeast, and Midwest regions of Brazil) also had increased odds of treatment default relative to the lower burden South and Southeast regions. As inadequately treated cases have the potential to contribute to onward transmission, this finding suggests that enhanced efforts to improve treatment completion in these communities could have the potential to contribute to disease control in the most affected regions. Additionally, our findings indicate that self-identification as having black ethnicity as well as markers of deprivation, related to income, access to electricity, and household crowding, were associated with higher odds of MDT default.

These important findings advance on prior research by indicating that individuals living in precarious socioeconomic conditions are not only at increased risk of leprosy infections [19, 20], but also they have an increased risk of treatment default following diagnosis. Few published studies have investigated factors associated with leprosy treatment default [7,16,21,22]. Factors suggested as barriers to adherence include poor household conditions, alcohol use, lack of knowledge about the disease and MB subtype [6, 7, 17]. In addition, a systematic review pointed to the need for more robust evaluations in this field, approaching regional particularities, since these associated factors may vary depending on the study location [9].

The largest previous study conducted in Brazil included 79 municipalities at high risk for leprosy transmission located in the Midwest region [6]. This study found that only low familial income (i.e., less than the current minimum wage) and reduced number of rooms (i.e., less than 3 per household) were associated with treatment default [7]. Our study provided important new evidence that geographic (i.e., region of residence), socioeconomic (i.e., black ethnicity) and household conditions (i.e., access to electricity)—factors well established as determinants of leprosy transmission [19, 23]—may also be associated with defaulting from MDT.

Evidence from the literature on socioeconomic factors associated with treatment default in other high leprosy burden countries is also scarce. In a study conducted in Nepal, most defaulters from MDT were illiterate, labourers and belonged to low-income families [21]. Another study, based in India, found an association of literacy status, per capita income and socioeconomic position with leprosy treatment outcomes. Higher default rates were evident among individuals that only completed primary education, had low per capita income, and belonged to the most deprived social classes [22]. In our study, the higher default rates among low income individuals might suggest the great financial impact of leprosy diagnosis and treatment on the affected households [24].

Our data also showed that living in households with informal lighting or no electricity was strongly associated with treatment default, mainly in the North region. Despite having adequate coverages of electricity, rural electrification of Brazil has not yet reached 100% [25]. Lack of access to electricity is an indicator of extreme poverty in the rural population. The use of irregular or informal sources of home lighting in peri-urban and urban areas also reflects socioeconomic deprivation [25, 26] and may be a marker for poor access to the healthcare system.

Consistent with previous research [7, 14, 15], our findings showed higher probabilities of default associated with geographic (residence in the North region) and socioeconomic factors (black ethnicity and low income) in individuals classified as MB leprosy, when compared to PB forms. With regards to the higher rates of default in MB leprosy cases, the longer duration of treatment for these patients may present an additional barrier to treatment adherence [7, 17].

Treatment default represents one of the most relevant obstacles to controlling chronic infectious diseases that require long-term treatment, such as leprosy [6]. A mathematical modelling investigation indicated that non-compliance to MDT and relapse of leprosy might have a negative impact on leprosy eradication, leading to an increase in disease prevalence and related deaths worldwide [27]. For the year 2017, Brazil was the country reporting the highest number of relapses (1734) to WHO [3]. Individuals classified as defaulters are at high risk of relapses and might have a higher chance of developing resistance to leprosy drugs, representing obstacles to this disease control [5, 9].

Among the main interventions to achieve leprosy control, the WHO recommends the strengthening of social and financial support with a focus on underserved populations, along with the use of a shorter and uniform regimen for all types of leprosy [5]. The use of a uniform multidrug therapy (U-MDT) regardless of any type of classification has been pointed out as the best option to halve treatment duration for MB patients (from 12 to 6 months) which could potentially decrease MDT default [28].

The strengths and limitations of this study should be stated. By linking nationally collected data on leprosy to socioeconomic information collected from more than 114 million individuals residing in all regions of Brazil, this study had an unprecedented sample size of leprosy cases with which to explore risk factors for leprosy default. Additionally, the inclusion of more than 20,000 cases enabled us to conduct stratified analyses and confirm that the associations were generally robust across leprosy subtypes and geographic regions. Importantly, this analysis also highlighted new factors associated with leprosy treatment default that have not previously been investigated (i.e. geographic location, ethnicity and household living conditions) in Brazil, the country with the second highest burden of leprosy worldwide [3].

On the other hand, this study also has limitations. First, as our data were collected routinely and not primarily for research purposes, 16.1% (3,848/23,911) of the linked individuals were excluded from the final analyses for having missing data. Second, we were unable to explore other determinants of default, such as characteristics of health services, individuals’ knowledge about the disease, and psychosocial and clinical factors, as these data were not available in our database. Qualitative assessment could provide a better understanding about the influence of these aspects in treatment completion of leprosy patients, as evidenced by a larger study conducted in Nepal aiming to understand people’s coping, help-seeking and adherence behaviour [29]. Third, although unlikely for most analysed socioeconomic characteristics, variables such as education and work might have changed in the time gap between the date of entry in the cohort and leprosy diagnosis. Finally, the generalizability of our results are restricted to individuals enrolled in CadÚnico, which represents approximately the poorest half of Brazilians who have registered for the national social protection programs. Although our findings may not be applicable to all leprosy cases in Brazil, it is likely that the point estimates of the associations between the indicators of deprivation and leprosy treatment default could be more pronounced if the full population of Brazil was included in the study.

Based on the study findings, we can conclude that poor socioeconomic conditions may constitute obstacles to leprosy treatment compliance. We also highlighted a remarkable association between black ethnicity and leprosy treatment default. However, the overall evidence on the correlation between ethnic background and leprosy is limited [30], which point to the need for further research. Our results also showed striking evidence on association of geographic and socioeconomic characteristics with treatment defaulting among MB leprosy individuals, who are the most important source of this disease transmission [2].

Decreasing default rates from MDT treatment has the potential to reduce the occurrence of relapses and physical disabilities and, by decreasing the infectious reservoir, may ultimately contribute to the goal of leprosy elimination. An integrated approach is needed, including actions on social determinants of leprosy and the adoption of full access to uniform treatment regimens for all PB and MB patients [5, 28], irrespective of material wealth. Other aspects that influence treatment default of leprosy cases, including distance from household to health service, adverse events/toxicity and mainly patient understanding the importance of correct treatment for cure should be better investigated. In addition to early diagnosis and prompt chemotherapy, social policies that reach the poor also at great risk of leprosy has been appointed about 100 years ago as a key strategy playing an important role and constituting a priority strategy to achieve leprosy control [31].

## Supporting information

S1 Checklist STROBE checklist. (DOC)
