# Correction: Geographic and socioeconomic factors associated with leprosy treatment default: An analysis from the 100 Million Brazilian Cohort

**DOI:** 10.1371/journal.pntd.0008723

**Published:** 2020-09-01

**Authors:** Kaio Vinicius Freitas de Andrade, Joilda Silva Nery, Julia Moreira Pescarini, Anna Ramond, Carlos Antônio de Souza Teles Santos, Maria Yury Ichihara, Maria Lucia Fernandes Penna, Elizabeth B. Brickley, Laura C. Rodrigues, Liam Smeeth, Mauricio L. Barreto, Susan Martins Pereira, Gerson Oliveira Penna

There is an error in the article XML causing the second author’s name to be indexed incorrectly. The name should be indexed as Nery JS. There is an error in the article XML causing the third author’s name to be indexed incorrectly. The name should be indexed as Pescarini JM. The correct citation is: de Andrade KVF, Nery JS, Pescarini JM, Ramond A, de Souza Teles Santos CA, Ichihara MY, et al. (2019) Geographic and socioeconomic factors associated with leprosy treatment default: An analysis from the 100 Million Brazilian Cohort. PLoS Negl Trop Dis 13(9): e0007714. https://doi.org/10.1371/journal.pntd.0007714

There are errors in the article XML causing the seventh, eighth, ninth, eleventh, twelfth, and thirteenth authors’ names to be indexed incorrectly. The names should be indexed as Penna MLF, Brickley EB, Rodrigues LC, Barreto ML, Pereira SM, and Penna GO, respectively.
