# Supplementary material for: Geographic and socioeconomic factors associated with leprosy treatment default: An analysis from the 100 Million Brazilian Cohort
Source: PLoS Negl Trop Dis. 2019 Sep 6;13(9):e0007714. doi: 10.1371/journal.pntd.0007714 (PMC6750604; doi:10.1371/journal.pntd.0007714)
Supplement: S1 Checklist — (DOC) [file pntd.0007714.s001.doc]

STROBE Statement

Checklist of items that should be included in reports of ***cohort studies***

**Manuscript title:** Geographic and socioeconomic factors associated with leprosy treatment default: An analysis from the 100 Million Brazilian Cohort

|  | Item No | Recommendation | Section and paragraph number |
| --- | --- | --- | --- |
| **Title and abstract** | 1 | (*a*) Indicate the study’s design with a commonly used term in the title or the abstract | Title page (p. 1) |
|  |  | (*b*) Provide in the abstract an informative and balanced summary of what was done and what was found | Abstract section  (p. 2) |
| Introduction | | |  |
| Background/rationale | 2 | Explain the scientific background and rationale for the investigation being reported | Introduction section (p. 4)  Paragraphs 1-4 |
| Objectives | 3 | State specific objectives, including any prespecified hypotheses | Introduction section (pp. 4-5)  Paragraph 5 |
| Methods | | |  |
| Study design | 4 | Present key elements of study design early in the paper | Methods section (p. 5)  Study design subsection  Paragraphs 1 and 2 |
| Setting | 5 | Describe the setting, locations, and relevant dates, including periods of recruitment, exposure, follow-up, and data collection | Methods section  Study population subsection (pp. 5-6)  Paragraph 1 |
| Participants | 6 | (*a*) Give the eligibility criteria, and the sources and methods of selection of participants. Describe methods of follow-up | Methods section  Study population subsection (pp. 5-6)  Paragraph 1 |
|  |  | (*b*) For matched studies, give matching criteria and number of exposed and unexposed | NA |
| Variables | 7 | Clearly define all outcomes, exposures, predictors, potential confounders, and effect modifiers. Give diagnostic criteria, if applicable | Methods section  Conceptual model subsection (p. 6)  Paragraphs 1-3 |
| Data sources/ measurement | 8* | For each variable of interest, give sources of data and details of methods of assessment (measurement). Describe comparability of assessment methods if there is more than one group |  |
| Bias | 9 | Describe any efforts to address potential sources of bias |  |
| Study size | 10 | Explain how the study size was arrived at | Methods section  Study population subsection (p. 6)  Fig 1 |
| Quantitative variables | 11 | Explain how quantitative variables were handled in the analyses. If applicable, describe which groupings were chosen and why | Methods section  Conceptual model subsection (p. 6)  Paragraph 1 |
| Statistical methods | 12 | (*a*) Describe all statistical methods, including those used to control for confounding | Methods section  Statistical analysis subsection (p. 7) Paragraphs 1-4 |
|  |  | (*b*) Describe any methods used to examine subgroups and interactions | Methods section  Statistical analysis subsection (p. 7) Paragraph 3 |
|  |  | (*c*) Explain how missing data were addressed | Methods section  Study population subsection (p. 5-6)  Paragraph 1 |
|  |  | (*d*) If applicable, explain how loss to follow-up was addressed | Methods section  Study population subsection (p. 5-6)  Paragraph 1 |
|  |  | (*e*) Describe any sensitivity analyses | Methods section  Statistical analysis subsection (p. 7) Paragraph 3 |
| Results | | |  |
| Participants | 13* | (a) Report numbers of individuals at each stage of study—eg numbers potentially eligible, examined for eligibility, confirmed eligible, included in the study, completing follow-up, and analysed | Methods section  Study population subsection (p. 6)  Fig 1 |
|  |  | (b) Give reasons for non-participation at each stage |  |
|  |  | (c) Consider use of a flow diagram |  |
| Descriptive data | 14* | (a) Give characteristics of study participants (eg demographic, clinical, social) and information on exposures and potential confounders | Results section (p. 8)  Paragraph 1 |
|  |  | (b) Indicate number of participants with missing data for each variable of interest | Methods section  Study population subsection (p. 6)  Fig 1 |
|  |  | (c) Summarise follow-up time (eg, average and total amount) | Results section (p. 8)  Paragraph 1 |
| Outcome data | 15* | Report numbers of outcome events or summary measures over time | Results section (p. 8)  Paragraph 1 |
| Main results | 16 | (*a*) Give unadjusted estimates and, if applicable, confounder-adjusted estimates and their precision (eg, 95% confidence interval). Make clear which confounders were adjusted for and why they were included | Results section (pp. 9-11)  Paragraphs 2-5 |
|  |  | (*b*) Report category boundaries when continuous variables were categorized | Results section (p. 8)  Table 1 |
|  |  | (*c*) If relevant, consider translating estimates of relative risk into absolute risk for a meaningful time period | NA |
| Other analyses | 17 | Report other analyses done—eg analyses of subgroups and interactions, and sensitivity analyses | Results section (p. 12)  Paragraphs 6-9 |
| Discussion | | |  |
| Key results | 18 | Summarise key results with reference to study objectives | Discussion section (p. 13)  Paragraph 1 |
| Limitations | 19 | Discuss limitations of the study, taking into account sources of potential bias or imprecision. Discuss both direction and magnitude of any potential bias | Discussion section (p. 15)  Paragraph 10 |
| Interpretation | 20 | Give a cautious overall interpretation of results considering objectives, limitations, multiplicity of analyses, results from similar studies, and other relevant evidence | Discussion section (pp. 13-15)  Paragraphs 2-8 |
| Generalisability | 21 | Discuss the generalisability (external validity) of the study results | Discussion section (p. 15)  Paragraphs 10-11 |
| Other information | | |  |
| Funding | 22 | Give the source of funding and the role of the funders for the present study and, if applicable, for the original study on which the present article is based | Funding section (p.19)  Paragraph 1 |
